# T2-mapping MRI evaluation of patellofemoral cartilage in patients submitted to intra-articular platelet-rich plasma (PRP) injections

**DOI:** 10.1007/s11547-021-01372-6

**Published:** 2021-05-18

**Authors:** Flavia Cobianchi Bellisari, Luigi De Marino, Francesco Arrigoni, Silvia Mariani, Federico Bruno, Pierpaolo Palumbo, Camilla De Cataldo, Ferruccio Sgalambro, Nadia Catallo, Luigi Zugaro, Ernesto Di Cesare, Alessandra Splendiani, Carlo Masciocchi, Andrea Giovagnoni, Antonio Barile

**Affiliations:** 1 Department of Biotechnological and Applied Clinical Sciences, University of L’Aquila, Via Vetoio, 1, 67100 L’Aquila, Italy; 2 Department of Radiologic Sciences, Azienda Ospedaliero Universitaria Ospedali Riuniti, Università Politecnica Delle Marche, Ancona, Italy; 3 Department of Health Sciences, University of L’Aquila, L’Aquila, Italy; 4 Radiology Department, S. Salvatore Hospital, L’Aquila, Italy

**Keywords:** T2 mapping, Articular cartilage, Knee, MRI, PRP, Joint injections

## Abstract

This study evaluated the ability of T2 mapping magnetic resonance imaging at 3 T, in addition to morphological sequences, to assess efficacy of platelet-rich plasma (PRP) injections, characterizing qualitatively and quantitatively the grade of knee cartilage repair in patients with patellofemoral chondropathy. We retrospectively studied 34 patients (22 men, 12 women, mean age 41.8 years, including 22 men) with patellofemoral knee chondropathy, who underwent intra-articular PRP injections and completed a clinical and instrumental follow-up. As control group, we evaluated 34 patients who underwent non-operative therapy. All patients were submitted to clinical (using VAS and WOMAC index) and imaging studies with 3 T magnetic resonance with cartilage analysis with T2 mapping sequences for cartilage analysis before and after treatment. In the study group, mean pre-treatment T2 relaxation time values were 44.2 ± 2.5 ms, considering all articular cartilage compartments, with significant reduction at the follow-up (*p* < 0.001). At the index compartment, mean pre-treatment T2 relaxation times values were 47.8 ± 3.6 ms, with statistically significant reduction at the follow-up (*p* < 0.001). Evaluation of focal cartilage lesions reported pre-treatment mean T2 value of 70.1 ± 13.0 ms and post-treatment mean value of 59.9 ± 4.6 ms (*p* < 0.001). From a clinical point of view, the pre-treatment WOMAC and VAS scores were 18.3 ± 4.5 and 7 (IQR:6–7.2), respectively; the post-treatment values were 7.3 ± 3.2 and 2 (IQR: 1.7–3.0), respectively (*p* < 0.001). In the control group, despite clinical improvement, we didn’t find significant T2 values change during the follow-up period. In conclusion, T2 mapping is a valuable indicator for chondropathy and treatment-related changes over time.

## Introduction

Articular cartilage damage due to overuse or trauma is common in the knee joint. Chondral alterations progress slowly, and clinical manifestations appear late in the process toward osteoarthritis (OA) [1–3]. The lack of early biomarkers elicits the development of valuable imaging methods [4]. Magnetic resonance imaging (MRI) is the modality of choice for diagnosing degenerative changes of knee toward OA, even if it can give limited information to detect early stages of OA and subtle changes in response to therapy [5–11].

The initial cartilage changes in OA include proteoglycan loss and degeneration of the collagen network, causing increased mobility of water and increased water content [12, 13]. In recent years, several quantitative advanced MRI techniques have emerged and have been proved to be feasible and reproducible for the evaluation of these biochemical chondral changes that occur before morphological alterations [14–19]. Among these imaging techniques, cartilage T2 relaxation mapping is a well-established analysis for the ultrastructural evaluation of the articular cartilage collagen matrix [20].

Intra-articular injection of platelet-rich plasma (PRP) has been broadly considered for cartilage repair, as it could enhance matrix synthesis thanks to the properties of its growth factors (mostly platelet-derived growth factor (PDGF) and transforming growth factor-beta (TGF-beta) [21–23]. However, despite the extensive literature confirming the positive clinical outcome in terms of pain and functionality improvement, there is scarce evidence demonstrating imaging results of the anabolic cartilage effects of PRP [24–26].

Our study aimed to assess the ability of T2 mapping sequences, in addition to morphological ones, to evaluate the efficacy of PRP intra-articular injections characterizing qualitatively and quantitatively the grade of knee cartilage repair in patients with patellofemoral chondropathy.

## Materials and methods

### Patients

The study protocol, approved by Institutional Internal Review Board, was designed as a retrospective study. Informed consent was obtained before any study-related procedure.

We retrospectively evaluated 85 patients who underwent intra-articular PRP injections at our institution between January 2015 and December 2019. Inclusion criteria included the following: age < 55 years; body mass index (BMI) < 30; availability of complete clinical report at baseline and up to 12 months after treatment; availability of 3 T MR examinations, including T2 mapping sequences at baseline and up to 12 months after treatment. Exclusion criteria were incomplete intra-articular PRP treatment sessions, surgical or other mini-invasive knee treatments, major knee trauma that occurred during the follow-up period. Thirty-four patients (22 males, 12 females, mean age 41.8 ± 8.9 years, range 22–54) met the aforementioned criteria and represented our final study population (Table 1).

**Table 1:**
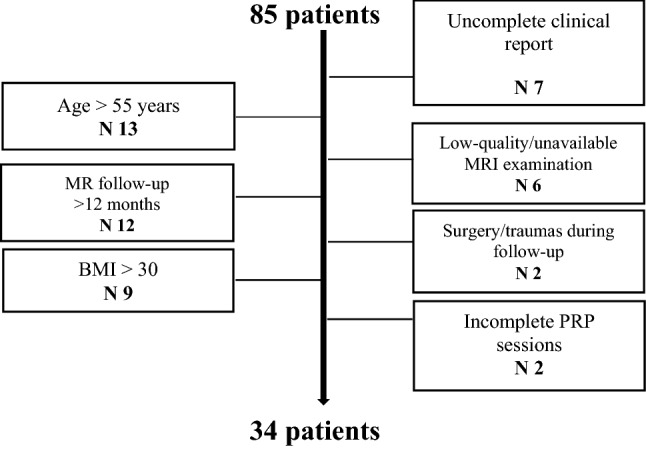
Flow chart of patient screening and study population selection

As control group, we selected 34 patients matching pairwise for age, sex, BMI, chondropathy severity who underwent conservative treatment.

### MRI protocol

All MRI examinations were performed on a 3 T MR scanner (MR 750 W, GE Healthcare), with a dedicated 16-channel knee coil (GEM flex coil). The imaging protocol included a T1-weighted sequence on sagittal plane (FOV 160 × 160 mm, TR/TE/NEX 525/min full/2, matrix 320 × 224, slice thickness 4 mm, gap 0.4 mm), T2-weighted sequence on axial plane (FOV 160 × 160 mm, TR/TE/NEX 6800/102/2, matrix 320 × 224, slice thickness 4 mm, gap 0.4 mm), T2 fat-saturated sequence on coronal plane (FOV 160 × 160 mm, TR/TE/NEX 6800/102/2, matrix 320 × 224, slice thickness 4 mm, gap 0.4 mm) and proton density (PD) sequences on sagittal plane (FOV 160 × 160 mm, TR/TE/NEX 1640/35/2, matrix 320 × 224, slice thickness 4 mm, gap 0.4 mm). T2 mapping was evaluated through a multi-echo spin-echo (SE) sequence with 8 echoes, acquired on axial plane, covering the complete patella, with the centre of the patella in the middle of the slab (FOV 160 × 160 mm, TR/TE 1000/7.6, 15.1, 22.7, 30.3, 37.8, 45.5, 52.9 and 60.5 ms, matrix 320 × 224, slice thickness 3 mm, gap 0 mm, bandwidth 228 kHz). The total acquisition time was 6:30 min.

### Image analysis

All baseline and follow-up MR images were analysed, after anonymization and randomization, by two experienced musculoskeletal radiologists (5 and 25 years of experience, respectively), blinded to the matching of the subjects and the timing of the examinations.

#### Morphological analysis

Semiquantitative analysis was assessed through morphological standard MR sequences using a modified whole-organ magnetic resonance score (WORMS) [27]. This modified score included the evaluation of four articular compartments (medial and lateral femoral condyle, medial and lateral patella) and analysis of the following parameters: cartilage signal intensity and morphology, subchondral bone marrow oedema, subchondral cysts, synovitis/joint effusion [28]. To evaluate cartilage signal intensity and morphology, we used an eight-point scale as follows: 0 = normal thickness and signal intensity; 1 = normal thickness but increased signal intensity on T2-weighted images; 2 = focal and partial-thickness focal defect < 1 cm in greatest width; 2.5 = full-thickness focal defect < 1 cm in greatest width; 3 = multiple areas of partial-thickness (grade 2.0) defects intermixed with areas of normal thickness, or a grade 2.0 defect wider than 1 cm but < 75% of the region; 4 = diffuse (≥ 75% of the region) partial-thickness loss; 5 = multiple areas of full-thickness loss (grade 2.5) or a grade 2.5 lesion wider than 1 cm but < 75% of the region; 6 = diffuse (≥ 75% of the region) full-thickness loss. Subchondral bone marrow oedema is considered as a non-well defined area of increased signal intensity in the epiphyseal marrow on fat-suppressed T2-weighted images. This feature was graded from 0 to 3 depending on extension of regional involvement: 0 = none; 1 =  < 25% of the region; 2 = 25% to 50% of the region; 3 =  > 50% of the region. Subarticular cysts were considered as areas of markedly increased signal in the subarticular bone with defined, rounded margins. Bone cysts were graded from 0 to 3 depending on extension of regional involvement, as for bone marrow abnormality: 0 = none; 1 =  < 25% of the region; 2 = 25% to 50% of the region; 3 =  > 50% of the region. Synovial thickening and joint effusion were graded collectively from 0 to 3 in terms of the estimated maximal distention of the synovial cavity: 0 = normal; 1 =  < 33% of maximum potential distention; 2 = 33–66% of maximum potential distention; 3 =  > 66% of maximum potential distention.

The articular compartment with the highest WORMS values was defined as the “index compartment”.

Further morphological assessment of focal cartilage lesions was performed using a modified Outerbridge classification that divides the pathological findings of chondromalacia into four grades. This classification was initially used for arthroscopic evaluation of patellar chondromalacia, then modified and extended to all chondral surfaces. The modified Outerbridge grading for joint cartilage breakdown depicts the following grades:

- Grade 0: normal cartilage;
- Grade I: hyperintense focal areas within normal contour (in arthroscopy cartilage is softening and swelling);
- Grade II: blister-like swelling/fraying of articular cartilage extending to the surface with a depth < 50% (in arthroscopy fragmentation and fissuring within soft areas of articular cartilage);
- Grade III: partial-thickness cartilage loss with focal ulceration with a depth > 50%;
- Grade IV: exposed subchondral bone with full-thickness defect

#### Qualitative analysis

**Fig. 1:**
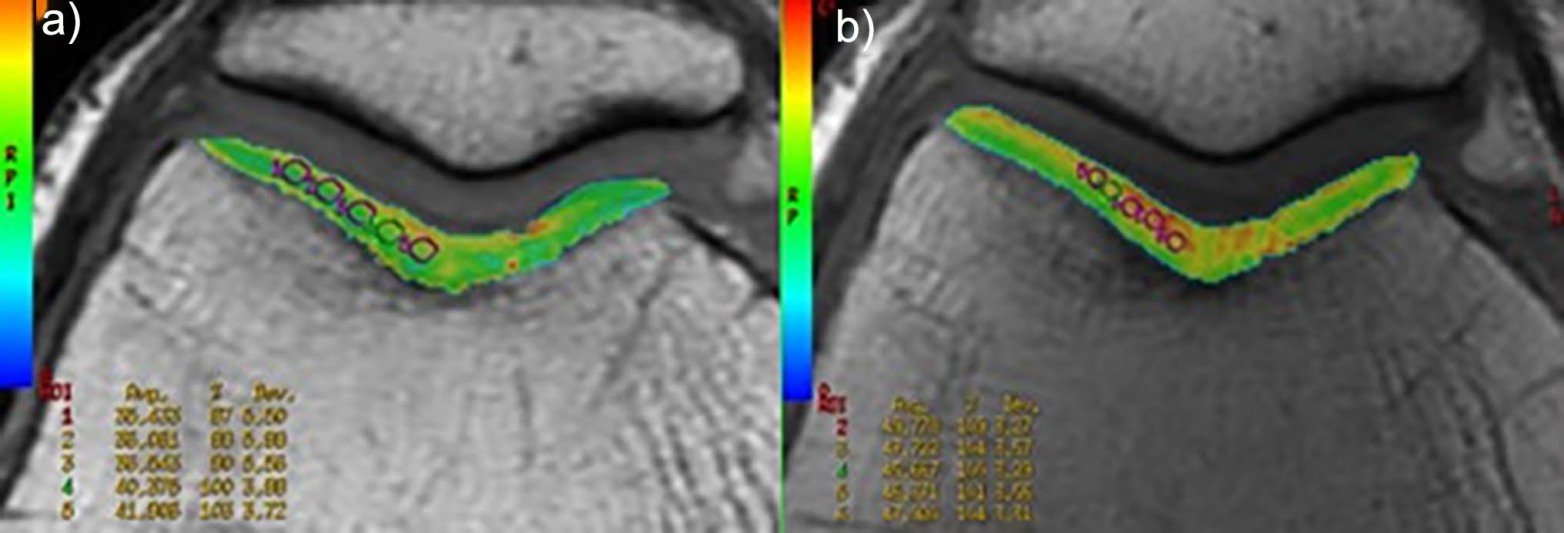
Imaging analysis of T2 mapping sequences. In **a**, ROIs positioning on the trochlear compartment, on an axial colorimetric map slice. In **b**, ROIs positioning in an area of focal cartilage damage, as depicted by T2 mapping colour maps (increased red signal)

For T2 mapping evaluation (Fig. 1), using the same anatomical WORMS articular segmentation, we positioned 3–5 regions of interest (ROIs) in the lateral and medial compartment of the patella and femoral condyle, respectively, on three planes (basal, intermediate, apical). T2 relaxation times colour maps were further used to place 3–5 ROIs in the areas of focal cartilage damage. To minimize sampling errors, ROIs had fixed small dimensions (3mm^2^), and were placed on high magnification images, to avoid interfaces with synovial effusion and subchondral bone. Based on previously obtained data from healthy volunteers using the same MR scanner, we considered the range between 28.3 and 41.2 ms as normal reference values, also in line with previous literature findings [29].

### Clinical evaluation

Clinical data retrieved from clinical reports included assessment through the visual analogue scale (VAS) for pain and the Western Ontario and McMaster University (WOMAC) index for pain and functionality. The WOMAC index is a self-administered health status measure assessing pain, stiffness, and function in patients with OA of the hip or knee. We usually also consider, in the context of the clinical assessment, BMI and grade of physical activity, that were recorded.

### Treatment protocol and technique

All patients of the study group were treated at our institution by the same experienced operator. For intra-articular injections of PRP, ultrasound (US) guidance using a linear probe (7–13 MHz) was used. The treatment protocol included three sessions, three weeks apart. Each treatment session included intra-articular administration of PRP (8–10 ml), previously activated with calcium chloride (1:20) and buffered with sodium chloride (1:10), using a 20-gauge needle. At the end of the procedure, all patients were required to avoid physical activities for the following two days, wearing a knee brace with 10 degrees of flexion.

### Statistical analysis

Data were collected, organized, and analysed through XLSTAT 2017: Data Analysis and Statistical Solution for Microsoft Excel (Addinsoft, Paris, France 2017). Intra- and inter-observer agreement was evaluated using the intraclass correlation coefficient (ICC). Qualitative variables were summarized as frequency and percentage, and the values of continuous variables were tested for normal distribution with Shapiro–Wilk’s test, and summarized as mean and standard deviation (SD) or median and interquartile range (IQR) according to their distribution. For each variable variation, pre vs. post values were calculated, and the treatment differences were compared with the Wilcoxon signed-rank test. All statistical tests were two-sided, and a level of statistical significance was set at *p* < 0.05.

## Results

The inter-observer agreement was excellent for both semiquantitative (ICC 0.93, 95% CI 0.85–0.94) and quantitative (ICC 0.92, 95% CI 0.86–0.93) analysis.

Mean follow-up interval between pre- and post-treatment clinical and imaging evaluation was 6.4 ± 1.9 months (range 4–12) after treatment.

### Study group

Morphological evaluation of patellofemoral cartilage (considering all articular compartments) showed pre-treatment mean modified WORMS score of 14 ± 3.4 (range 10.5–18); at the follow-up, mean values were 12.5 ± 2.4 (range 10–15), with a mean improvement of 10.5% (*p* < 0.001). Focussing on the index compartment, pre-treatment modified WORMS mean values were 4.5 ± 1.7 (range 3–9); at the follow-up, mean values were 4.0 ± 1.3 (range 3–7), with a mean improvement of 11% (*p* < 0.001) (Table 2).

**Table 2:** Pre- and post-treatment values of WORMS scores assessed for the global joint and for the index compartment

	WORMS score			
	Baseline	Post-treatment	Δ (%)	*p*
Global	14 ± 3.4	12.5 ± 2.4	10.5	*p* < 0.001
Index compartment	4.5 ± 1.7	4 ± 1.3	11	*p* < 0.001

We found 26 focal cartilage lesions. Based on Outerbridge grading, at baseline, we found 18 grade III lesions and 4 grade IV. At the follow-up, we registered 22 grade III lesions and 0 grade IV, with an improvement in 15.4% of patients (Table 3).

**Table 3:** Outerbridge classification: baseline and post-treatment values

	Outerbridge classification							
	Grade I (*n*)		Grade II (*n*)		Grade III (*n*)		Grade IV (*n*)	
	Baseline	Follow-up	Baseline	Follow-up	Baseline	Follow-up	Baseline	Follow-up
Study group	0	0	4	4	18	22	4	0
Control group	0	0	5	5	15	14	5	6

**Fig. 2:**
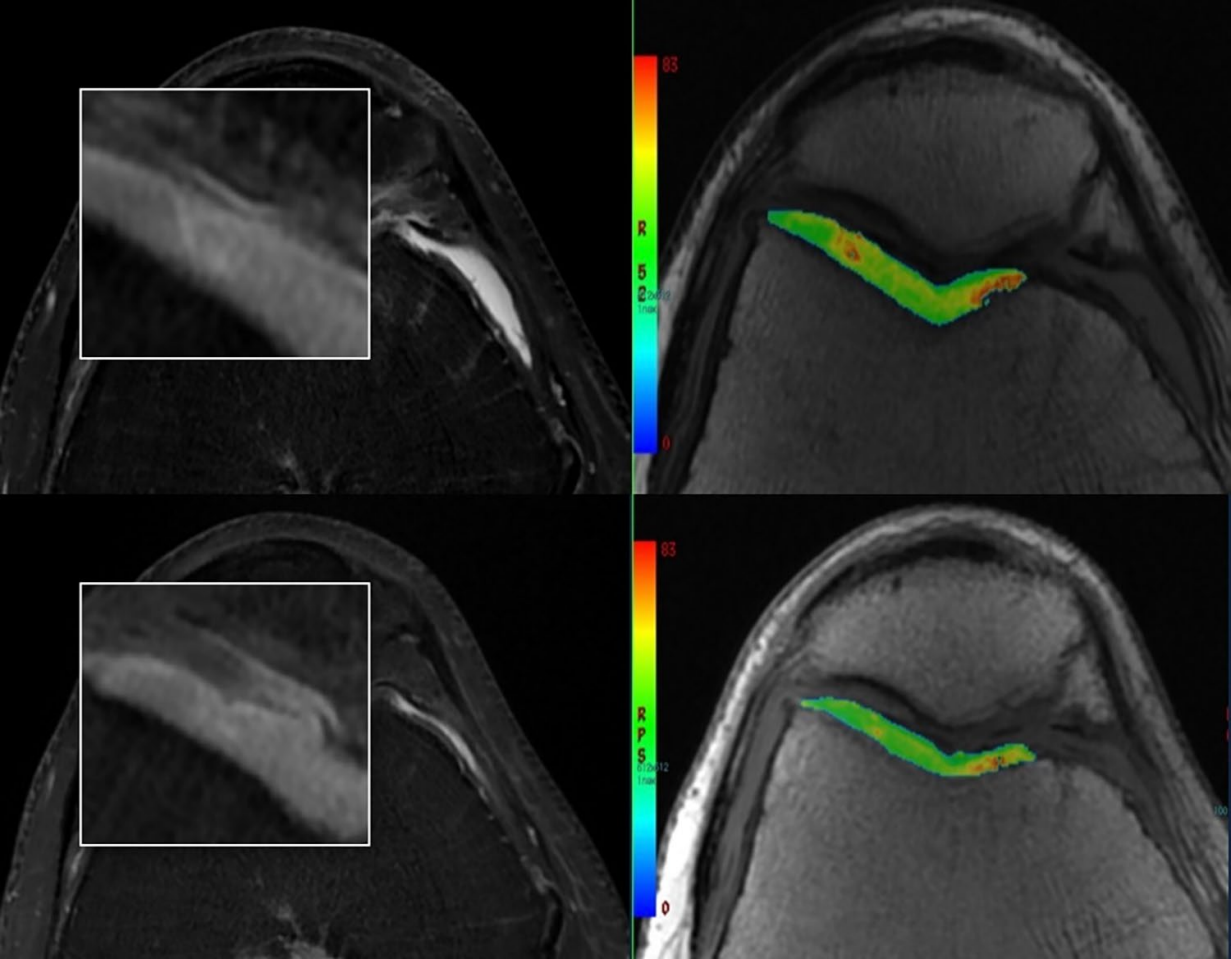
Upper row (baseline): axial PD with fat saturation showing a focal area of altered signal at the level of the lateral trochlear cartilage; quantitative analysis confirms higher T2 relaxation time values, consistent with collagen degradation and chondropathy. Lower row (6-month after PRP injections): axial PD with fat saturation and quantitative analysis showing lower T2 relaxation time values

Quantitative analysis (Fig. 2), considering all cartilage compartments, showed pre-treatment mean T2 relaxation times values of 44.2 ± 2.5 ms (range 39.49–49.37) and post-treatment mean values of 41.5 ± 2.5 ms (range 37.37–45.23), with a statistically significant difference (*p* < 0.001). At the index compartment, we found pre-treatment mean T2 values of 47.8 ± 3.6 ms (range 41.61–54.31). The index compartment was lateral inferior condyle in 12 patients, lateral superior condyle in 2 patients, medial inferior trochlea in 10 patients, medial intermediate trochlea in 4 patients, medial superior condyle in 2 patients, lateral intermediate patella in 2 patients, medial intermediate patella in 2 cases, and post-treatment mean values of 43.5 ± 3.8 ms (36.86–50.79), with a statistically significant difference (*p* < 0.001). Evaluation of focal cartilage lesions showed pre-treatment mean T2 values of 70.1 ± 13.0 ms (55.04–97.12) and post-treatment mean values of 59.9 ± 4.6 ms (52.72–65.22)(*p* < 0.001). We registered also mean T2 values of each compartment before and after treatment (Table 4).

**Table 4:** Baseline and post-treatment T2 relaxation time values (ms) of study group

	Pre-treatment	Post-treatment	Improvement	*p*
Global	44.2 ± 2.5 (49.37–39.49)	41.5 ± 2.5 (45.23–37.37)	− 6.0%	< 0.001
Index compartment	47.8 ± 3.7 (54.31–41.61)	43.5 ± 3.9 (50.79–36.86)	− 9.0%	< 0.001
Focal cartilage lesion	70.1 ± 13 (55.04–97.12)	59.9 ± 4.6 (65.22–52.72)	− 14.5%	< 0.001

	Medial patellar compartment	Lateral patellar compartment	Medial femoral condyle	Lateral femoral condyle
Pre-treatment	40.4 ± 3.8	40.1 ± 5.1	48.5 ± 3.1	47.6 ± 3.7
Post-treatment	37.6 ± 4.3	38.1 ± 4.2	44.7 ± 3.7	45.7 ± 2.9
*p* value	< 0.001	< 0.001	< 0.001	< 0.001

**Fig. 3:**
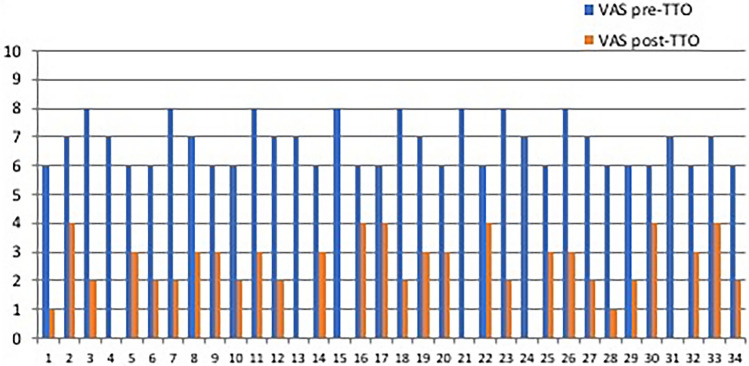
VAS: pre- and post-treatment marks for pain. The results show improvement with statistical significant rates. On x-axis number of people; on y-axis VAS values

**Fig. 4:**
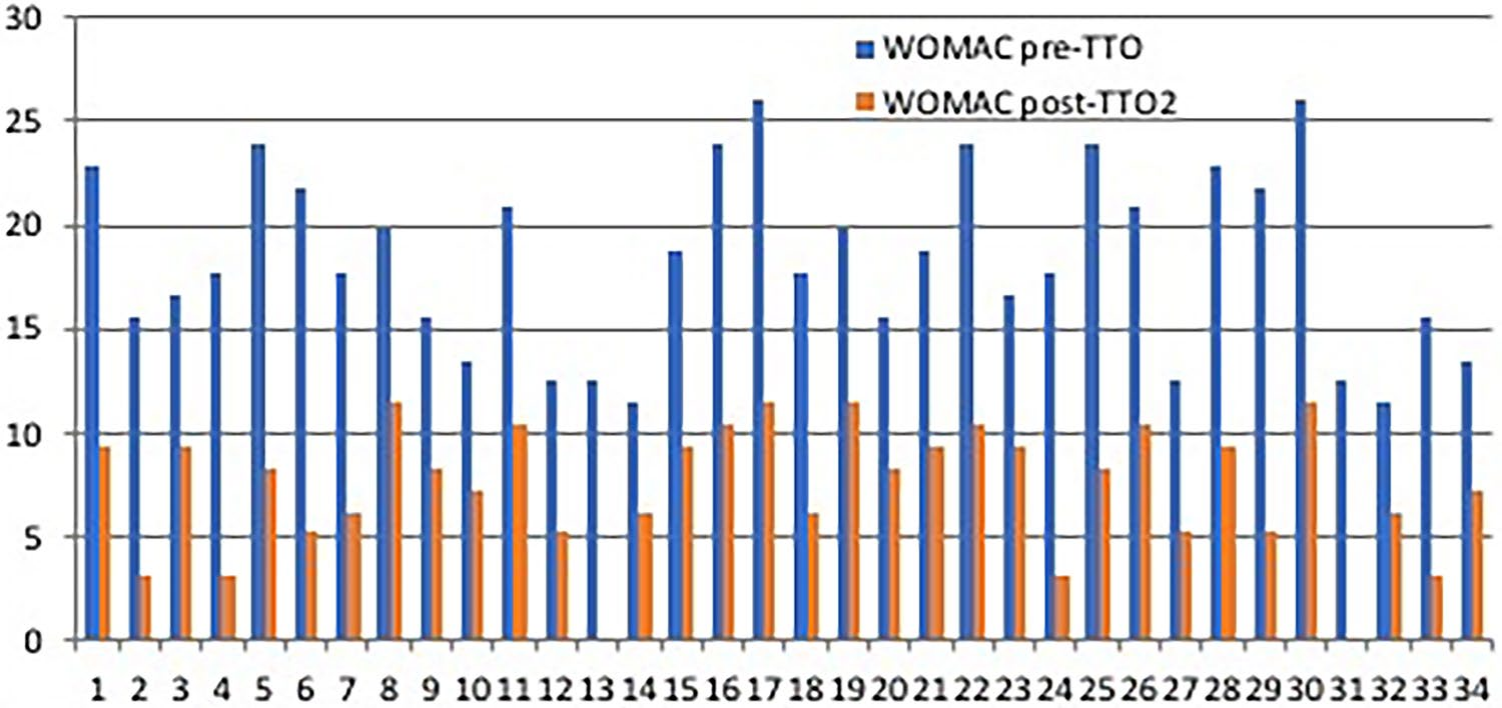
WOMAC: pre- and post-treatment marks for pain. The results show improvement with statistical significant rates. On x-axis number of people; on y-axis WOMAC values

From a clinical point of view (Fig. 3, Fig. 4), the pre-treatment values according to the WOMAC and the VAS scores were 18.3 ± 4.5 and 7 (IQR: 6.0–7.2); at the follow-up, a significant improvement was observed in both cases. The post-treatment values were 7.3 ± 3.2 and 2 (IQR: 1.7–3.0), respectively. Pre- and post-treatment differences were likewise statistically significant in both cases (*p* < 0.001).

### Control group

Morphological evaluation of patellofemoral cartilage (considering all articular compartments) showed pre-treatment mean modified WORMS score of 15 ± 2.9(range 11.6–19); at the follow-up, mean values were 15.5 ± 2.6 (range 11–17), with a mean improvement of 3.3% (*p* = 0.132). Focussing on the index compartment, pre-treatment modified WORMS mean values were 4.9 ± 1.5 (range 4–10); at the follow-up, mean values were 4.8 ± 1.5 (range 4–9), with a mean improvement of 2.04% (*p* = 0.142) (Table 5).

**Table 5:** Pre- and post-treatment values of WORMS scores of control group

	WORMS score			
	Baseline	Post-treatment	Δ (%)	*p*
Global	15 ± 2.9	15.5 ± 2.6	3.3	0.132
Index compartment	4.9 ± 1.5	4.8 ± 1.5	2	0.142

We found 25 focal cartilage lesions. Based on Outerbridge grading, at baseline, we found 5 grade II lesions, 15 grade III and 5 grade IV. At the follow-up, we found 14 grade III and 6 grade IV (worsening in 0.3% of patients) (Table 3).

Quantitative analysis, considering all cartilage compartments, showed pre-treatment mean T2 relaxation times values of 43.2 ± 1.8 ms (range 47.43–38.50) and post-treatment mean values of 43.1 ± 2.1 ms (range 46.22–38.35), without reaching a statistically significant difference (*p* = 0.121). At the index compartment, we found pre-treatment mean T2 values of 46.9 ± 3.5 ms (range 53.2–42.10). The index compartment was lateral inferior condyle in 11 patients, lateral superior condyle in 3 patients, medial inferior trochlea in 8 patients, medial intermediate trochlea in 6 patients, medial superior condyle in 3 patients, lateral intermediate patella in 2 patients, medial intermediate patella in 1 cases, and post-treatment mean values of 47.1 ± 3.8 ms (51.79–37.91), without reaching a statistically significant difference (*p* = 0.105). Evaluation of focal cartilage lesions showed pre-treatment mean T2 values of 72.3 ± 15 ms (56.04–97.12) and post-treatment mean values of 72.6 ± 4.2 ms (65.22–52.72)(*p* = 0.132). We registered also mean T2 values of each compartment before and after treatment (Table 6).

**Table 6:** Baseline and post-treatment T2 relaxation time values (ms) of control group

	Pre-treatment	Post-treatment	Percentage of improvement (%)	*p*
Global cartilage	43.2 ± 1.8 (47.43–38.50)	43.1 ± 2.1 (46.22–38.35)	− 0.2	0.121
Index compartment	46.9 ± 3.5 (53.2–42.10)	47.1 ± 3.8 (51.79–37.91)	− 0.4	0.105
Focal cartilage lesion	72.3 ± 15 (56.04–97.12)	72.6 ± 4.2 (65.22–52.72)	0.4	0.132

	Medial patellar compartment	Lateral patellar compartment	Medial femoral condyle	Lateral femoral condyle	*p*
Pre-treatment	42.5 ± 1.5	41.2 ± 5.5	46.1 ± 3.5	46.6 ± 3.9	< 0.001
Post-treatment	42.2 ± 3.9	41.1 ± 4.5	45.9 ± 3.9	46.1 ± 3.1	< 0.001

From a clinical point of view, the pre-treatment values according to the WOMAC and the VAS scores were 17.5 ± 5.1 and 8 (IQR: 5.0–7.5); post-treatment values were 15.6 ± 4.9 and 6 (IQR: 5.3–6.9), respectively. Pre- and post-treatment differences were not statistically significant in both cases.

## Discussion

Chondral lesions represent a clinical-diagnostic challenge [30–33]. Data of 2005 counted in the USA about 27 million people suffering from chondropathy for what concerns knee joint, and about 450,000 knee arthroplasties were performed, with a projected growth to 3.5 million by 2030 [34].

Thanks to the progress in molecular biology, several conservative therapies for early chondropathy have emerged [35]. Among new regenerative methods, intra-articular PRP therapy plays an important role as it may favour the healing process and tissue regeneration [36]. PRP contains high concentrations of growth factors, including transforming growth factor-b, insulin-like growth factor, platelet-derived growth factor, vascular endothelial growth factor, and epidermal growth factor, that regulate chondral homeostasis, with a positive effect on healing process as on chondrogenesis.

In vitro studies showed that PRP stimulates cellular proliferation and chondral matrix by chondrocytes. PRP, added to culture medium, promotes porcine chondrocytes concentration and collagen and proteoglycan syntheses [37]. What is more, PRP has a positive effect on stem cell proliferation and expression of chondrogenic markers [38].

In vivo studies showed that PRP could downregulate catabolic processes of collagenases and have a positive effect on anabolic response to cartilage damage [39]. PRP also revealed a positive role in reducing inflammatory cascade in OA [40].

PRP showed a positive effect on cartilage repair and restoration after microfractures in studies based both on animals and humans [41, 42]. Moussa M. et al. [43] demonstrated the positive effect of PRP on chondrocytes, synovial and stem mesenchymal cells improving cell proliferation, extracellular matrix, and hyaluronic acid syntheses; PRP can also provide a bioactive structure in cartilage defects. Wang-Saegusa A. et al. [44] reported that, at a 6-month follow-up, intra-articular infiltration of autologous PRP in patients with OA of the knee has local benefits, reducing pain and restoring function, with consequent improvement of quality of life. Several studies compared hyaluronic acid and PRP injections, showing at 6-month follow-up better results with PRP in terms of clinical effects [45]; Sanchez M. et al. [46] registered that success rate for the pain sub-scale reached 33.4% for PRP group and 10% for HA group. Following the literature, our study confirms an overall clinical improvement at a short-medium term follow-up period, regardless of chondral lesion grade.

However, many questions remain unanswered regarding the efficacy of PRP, due to the heterogeneity of the studies and the PRP preparation and administration protocols.

A recent review [47] highlighted some variables in PRP preparations, analysing different parameters. First, the type of PRP, distinguishing pure platelet-rich plasma (P-PRP) and leukocyte platelet-rich plasma (L-PRP); second, different concentrations of platelet enrichment factor. What is more, there are several activation modalities for PRP (thrombin, calcium chloride, or mechanical trauma). Another variable is the use of different phases of centrifugation (single/double, manual, or automated methods of selective filtration procedures, closed or open circuits) with an influence on the number of platelets and growth factor concentration.

Moreover, there is still a lack of studies that evaluate MRI findings, either with standard or advanced (as T2 mapping) sequences to assess imaging evidences of PRP efficacy [48].

Several semiquantitative scales have been proposed for standard morphological evaluation of knee OA; KOSS (Knee Osteoarthritis Scoring System) [49], BLOCKS (Boston Leeds Osteoarthritis Knee Score) [50], the more recent MOAKS (MRI Osteoarthritis Knee Score) [51] have been widely used. WORMS has emerged to be the method of choice for assessment of bone marrow lesions and as a predictive factor of cartilage loss [52].

In our study, we used the WORMS, as this semiquantitative scoring method appears more reproducible and applicable to quantitative T2 mapping evaluation.

Regarding imaging analysis, we observed morphological improvement on the articular cartilage either globally or at the index compartment. Concerning joint cartilage focal lesions, we chose Outerbridge classification as it focuses on cartilage lesions. Our results show a reduction of the Outerbridge score in 15.4% of lesions after treatment.

T2 mapping is a novel molecular imaging technique developed to detect early stages of OA, as biochemical alterations precede the morphological ones [53]. In fact, the initial articular cartilage degenerative changes include proteoglycan loss and degeneration of the collagen network, which causes increased water mobility and water content. T2 relaxation time mapping can reflect these changes in cartilage ultrastructure [54]. As it has been recently reported, patients with anterior knee pain show high T2 mapping values [55]. Association between cartilage degeneration and T2 values has been demonstrated in several studies conducted on both animals [56] and humans [57, 58], and on histopathological findings in vitro [59, 60]. It emerged that high T2 values characterize articular cartilage of patients with risk factors for OA [61].

To fulfil a gap in the literature, we wanted to measure the difference of T2 values at baseline and after PRP injections in patients affected by patellofemoral chondropathy, in order to have a quantitative evaluation of PRP efficacy and cartilage healing process, also employing a colour-scale representation. We found that T2 mapping values showed a statistically significant improvement at short-medium term follow-up, either evaluating the cartilage compartments globally and at the index compartment. Focussing on focal cartilage lesions, T2 mapping values had a statistically significant reduction. This finding suggests that T2 relaxation time mapping could be a useful biomarker for therapy response, as it is a sensible technique that can detect subtle alterations that precede morphological changes.

It is still unanswered if T2 mapping could have a predictive value. Some recent studies [62–64] based on large courts, detected progression of OA degenerative changes, showing the predictive prognostic role of T2 mapping. Tratting S. et al. [55] assessed the quality of repair tissue of patients after GelrinC implantation in the femoral condyle, finding that low T2 values could mean implant incorporation; they used T2 values to assess quality and status of repaired cartilage.

In line with previous observations, we used a 3 T scanner; Wong et al. [65] reported that MRI at 3 T improved visualization of anatomical structures and improved diagnostic confidence compared to 1.5 T, resulting in significantly better sensitivity (75.7% vs. 70.6%), accuracy (88.2% vs. 86.4%) and correct grading of cartilage lesions of the knee (51.3% vs. 42.9%), using arthroscopy as a standard reference.

One of our main limitations is represented by the lack of a diagnostic reference standard (arthroscopy or histology), even if there are several studies comparing T2 mapping values with histological findings; further, the observation period is relatively short, and there are intrinsic limitations of a retrospective study.

Another weakness of our study is the lack of standardization of the management and treatments performed in the control group, due to the retrospective selection.

On the other hand, much of the strength of this study lies in the fact that we selected a homogeneous study population based on BMI (normal weight, range 18.5–24.9) and physical activity; moreover, being a single-centre study, PRP injections were performed by the same operators and PRP protocol preparation was stable (L-PRP).

## Conclusions

Our results confirm the positive clinical effect of PRP in patients affected by patellofemoral knee chondropathy. In addition to morphological sequences, 3 T MRI with T2 mapping is a valuable tool for the evaluation of cartilage water content and thus PRP injections efficacy. Further, characterization of the cartilage matrix integrity with T2 mapping may help in the prevention of disease progression by enabling the identification of individuals with early osteoarthritis who may benefit from treatment before irreversible morphologic changes occur.

## Data Availability

Not applicable.
